# Microcomputed Tomography Evaluation of Root Dentin Caries Prevention by Topical Fluorides and Potassium Iodide

**DOI:** 10.3390/s19040874

**Published:** 2019-02-20

**Authors:** Vernon Zander, Daniel Chan, Alireza Sadr

**Affiliations:** Department of Restorative Dentistry, University of Washington, Seattle, WA 98195, USA; zandev@uw.edu (V.Z.); dcnchan@uw.edu (D.C.)

**Keywords:** micro CT, mineral content, silver diammine fluoride, demineralization, root dentin, fluoride, potassium iodide

## Abstract

The mineral content of dental hard tissues has traditionally been measured by destructive tests such as transverse microradiography. Microfocus X-ray computed tomography (micro CT) has enabled non-destructive 3D assessment of tooth demineralization. This study compared the preventive effects of silver diammine fluoride (SDF) and potassium iodide (KI) in comparison with fluoride varnish. SDF has been known to arrest caries but darkens the tooth. KI creates a precipitate with SDF that reduces the discoloration, but its effects on SDF efficacy in terms of preventing demineralization of at-risk root dentin surfaces is unknown. Bovine root dentin blocks were randomly distributed into four groups and subjected to a pretreatment in each group (n = 8); Control: deionized water (DIW); F-Varnish: 5% sodium fluoride varnish: 38% Saforide; SDF+KI: SDF followed by saturated solution of KI in DIW. The treated dentin was subjected to 8 cycles of demineralization (pH 5) for 14 h and remineralization in artificial saliva (pH 7) for 10 h. Specimens were then scanned for 12 min using micro CT at 73 KV and 1012 µA with 8.3 μm resolution. The 3D images were analyzed in Amira software to calculate lesion depth (LD), surface layer mineral density (SL) and mineral loss (ΔZ) for each specimen. One-way ANOVA with Bonferroni posthoc showed that there was a statistically significant difference between Control and all three other groups for all parameters (P < 0.001), however, there was no statistical difference among F-Varnish, SDF and SDF+KI (P > 0.05). Single application of F-Varnish, SDF and SDF+KI showed comparable preventive effects against root dentin demineralization. Application of KI did not affect anti-demineralization properties of SDF in this study. Micro CT is a quick and effective method for objective and high-resolution characterization of dentin caries lesions.

## 1. Introduction

With continuing population expansion, and the decreasing availability of dentists to provide emergency care and restorative treatment [1], there is a growing need to find a preventive and minimally invasive treatment for treating dental coronal and root caries. The traditional restorative approach to treat caries can be both costly and invasive as the material and operating time increases. While several methods to treat caries exist, application of silver diammine fluoride (SDF) is one of the least invasive [2]. Other chemotherapeutic approaches have included the use of Chlorhexidine rinses, but the evidence is not conclusive as to how effective these rinses can be [3]. It has been shown that after a short time, oral flora can become resistant to the rinse, leading to a reduction in biofilm removal the more it is used. In contrast, one application of SDF could arrest caries and is not limited by an increasing resistance like chlorhexidine [4]. Patient compliance can also be an issue with rinses as the patient will need to remember to swish each day for the specified time. Over time patient compliance could decrease leading no noncompliance as opposed to SDF that can be applied once for a full effect on the carious dentin. 

SDF is a metal ammine complex of silver fluoride; the common formulation for dental application is 38% SDF which has 44,800 ppm fluoride ion and 255,000 ppm silver ions. SDF was originally developed in the 60s in Japan and has been used in many countries for decades. Since 2014, the FDA has approved SDF for use in the United States as a desensitizer and it has since proven to be a viable possibility to reduce caries.

SDF is a minimally invasive and preventive treatment that could help in providing care to those with dental fears as well as pediatric populations. Application is also simple, the solution is low-cost, and application does not require complex training of the health professionals. SDF has been suggested as a method to arrest or prevent caries in at-risk populations, such as the geriatric or socially deprived cases, which are more likely to develop caries and often wait until unbearable symptoms arise before seeking treatment [1,5,6]. Root caries seems to be an emerging and hard to treat issue in these populations. A special attention has also been paid to the potential of SDF to prevent caries on the exposed root dentin surfaces [7], which are highly susceptible to biofilm accumulation and rapid progress of demineralization through dentin. However, SDF will oxidize to a black color on exposure to light due to the formation of a silver oxide layer and as such, it can be an esthetic concern for use in adults, and occasionally the parents of children [8]. The addition of a supersaturated solution of potassium iodide (KI) can decrease the sharp black color and leave a more aesthetically pleasing result. However, the effect of this additive on the performance of SDF as a root caries prevention is not fully understood and needs to be researched further. 

Demineralization studies are the most basic types of tests required to predict the performance of preventive therapies in dentistry. The transverse microradiography (TMR) technique has been used extensively for mineral density measurement in cariology [9,10,11]. The technique relies on preparation of very thin physical cross-sections of specimens and imaging them under X-ray. These slices, commonly hard tissue slices about 100 µm-thick are not only fragile and a labor-intensive challenge to create, they limit data to a certain area in the specimen. Microfocus X-ray computed tomography (micro CT) is a high resolution 3D X-ray imaging technique that has evolved over the past few years along with advancements in computer science. Image analysis methods have been validated to measure TMR lesion parameters such as lesion depth (LD) and mineral loss (ΔZ) based on the CT values representing radiopacity for enamel [12], and modified for dentin lesions [13]. As a non-destructive method [14], specimens can be scanned whole, without needing special preparations. These samples can be scanned quickly and clearly, providing accurate data points. Not only can these samples be saved for later use, but when the sample is scanned whole there are hundreds of slices (a 3D volume) created as opposed to one cross-section.

Therefore, the aim of this study was to determine the demineralization-resistance of SDF and KI-treated bovine root dentin, compared to a standard 5% fluoride varnish using micro CT. The null hypothesis was that there was no difference in demineralization resistance of root dentin treated by water, fluoride varnish, SDF or SDF-KI.

## 2. Materials and Methods

### 2.1. Specimen Preparation

Freshly extracted bovine incisors were obtained from a local facility for this study. The animal protocols used in this work were evaluated and approved by the Institutional Animal Care and Use Committee (IACUC) of the University of Washington (Animal Welfare Assurance Approval number A3464-01) in compliance with state and federal regulations. The teeth were stored frozen (0 °C) until use. The incisors were collected from a local slaughterhouse. The roots were cleaned using water and a scalpel until the entire surface was free of organic material. The teeth were then cross-sectioned below the cementoenamel junction (CEJ) using a diamond blade (Isomet 11-4344, Buehler, Lake Bluff, IL, USA) attached to a low-speed precision saw (CL-50, Preciso, Taichung, Taiwan). The root was then attached to an acrylic block using cyanoacrylate adhesive (Model Repair II Blue; Dentsply-Sankin, Tochigi, Japan), and 32 dentin blocks approximately 6 × 7 × 3 mm^3^ were cut from the root sections. All of the blocks were then embedded in slow-setting epoxy resin (TotalBoat, Bristol, RI, USA) so that only the surface of the root was exposed. The exposed dentin was wet polished using #600 SiC paper (3M, St. Paul, MN, USA) to create a smooth and flat dentin surface. The exposed root surfaces were finally covered with nail varnish (Revlon, New York, NY, USA) so that two windows each 6 × 3 mm^2^ were exposed. The blocks were randomly assigned to one of the four experimental groups (n = 8). On each block, the nail-varnish covered root surface served as the sound reference, one exposed window, selected randomly, served as the internal control for the specimen while the other one was subjected to the pretreatment as follows.

### 2.2. Treatment Protocols

#### 2.2.1. Control

The treatment window was rinsed with deionized (DI) water for 30 s.

#### 2.2.2. F-Varnish

The protocol for application of 5% sodium fluoride varnish (Duraflor, Medicom, Augusta, GA, USA) followed the manufacturer’s instructions. The prepared root surfaces were dried with a paper towel and a microbrush dipped into the varnish so that 0.5 mL was applied and allowed to air dry.

#### 2.2.3. SDF

The protocol used for 38% Saforide (Bee Brand Medico Dental, Tokyo, Japan) was designed following the manufacturer’s instructions. Sections of root were dried using a paper towel, then 1-2 drops of SDF was added to a mixing well and applied with a microbrush for ten seconds. The specimens then sat for 1 minute and the excess SDF was removed via rinsing with DI water and air dried.

#### 2.2.4. SDF+KI

The protocol for the SDF+KI group was based on the methods used previously [15]. Sections of root were dried, then 1 drop of SDF was applied with a microbrush for 10 s. Immediately following the application of SDF, saturated KI solution composed of 1.44 g/mL KI (Sigma-Aldrich, St. Louis, MO, USA) in DI water was applied until a white-yellow precipitate was formed. The root surface was then rinsed with DI water and air-dried. 

### 2.3. De/Remineralization Cycles

Within 10 min after treatment, each prepared specimen in the experimental groups was subjected to demineralization and remineralization cycles of 14 h and 10 h, respectively, for 8 days at 37 °C. Demineralization solution was composed of 1.5 mM CaCl_2_, 0.9 mM KH_2_PO_4_ and 50 Acetic acid, adjusted to a pH of 5 with KOH [16], and remineralization solution was based on an artificial saliva composition with 1.5 mM CaCl_2_, 130 mM KH_2_PO_4_, 20 mM HEPES, adjusted to pH 7 using KOH [16]. The specimens were separately immersed in 5 mL of the solution in a multiwell culture plates. The solutions were exchanged daily. After each cycle, the block was rinsed with DI water for 30 s before it was returned to the plate for the next cycle.

### 2.4. Micro CT Imaging

**Figure 1:**
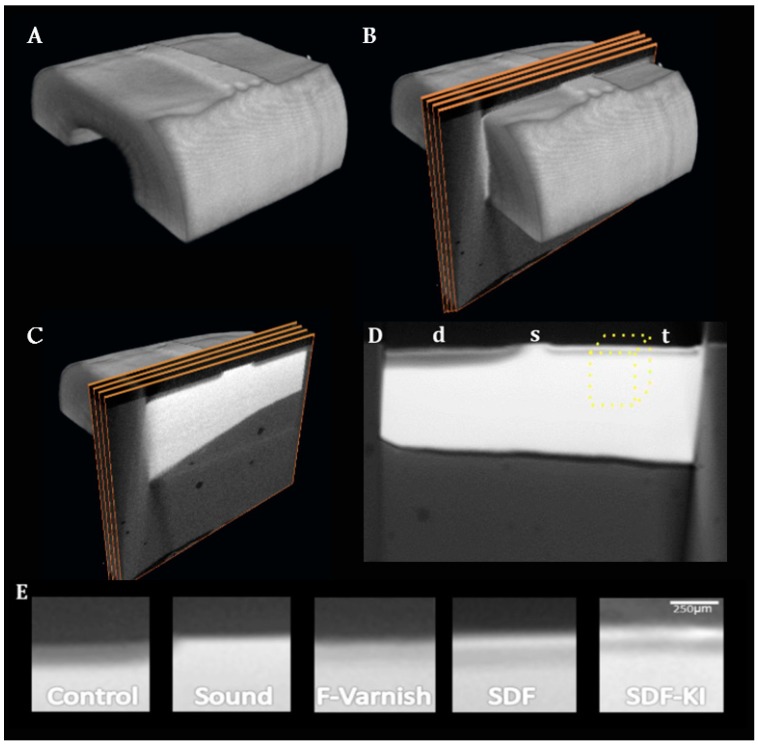
3D reconstruction of images in Amira software and the results. (**A**) a root dentin block specimen scanned; (**B**) 100 slices were then selected from the middle of each specimen and averaged to one image; (**C**) representative slices on the 3D image; (**D**) the resulting average image. The volume of interest on the treatment window is highlighted with the dotted outline. The demineralized internal control (d), sound (s) and treated (t) areas are distinctly visible; (**E**) representative average slices used to calculate mineral density in each group.

**Figure 2:**
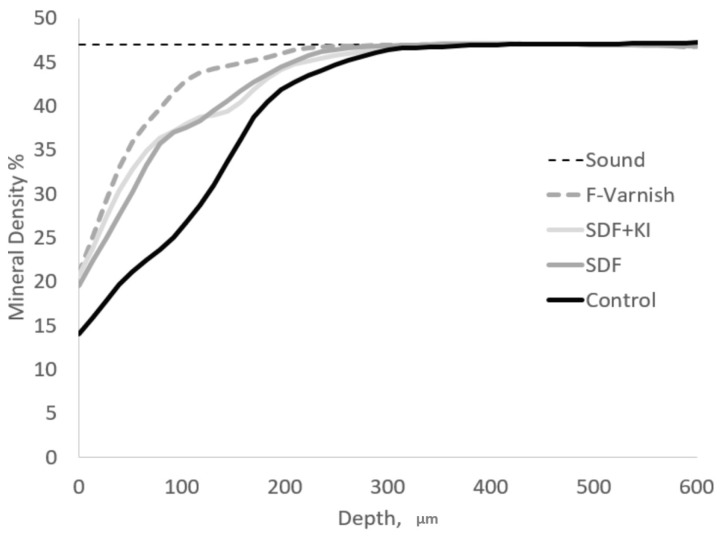
Micro CT mineral density vs. depth profile of representative specimens for all groups. Lesion depth (LD) represents the depth aspect from surface up to where the mineral density is within 5% of the sound tissue (48% in dentin). Mineral loss (ΔZ) represents the area over the curve for each mineral density profile.

The specimens were then scanned using an X5000 micro CT device (North Star Imaging, Irvine, CA, USA) using the following parameters; pixel size (actual resolution) of 8.3 μm, voltage 73 KV and 1012 µA. Each specimen was attached to a custom 3D-printed jig that would consistently hold all specimens in the imaging window at the center of rotation of the micro CT turntable using utility wax. The jig was then bolted to the stage and rotated for 360° at a resolution of 2048 by 2048 pixels per projection. The total integration time was 12 min. A moist paper towel was placed over the dentin block to prevent dehydration and demineralized collagen matrix shrinkage during each scan. The images were then reconstructed via the default system software, which accounted for beam hardening correction and geometry. A geometry tool had to be scanned with similar scan parameters as the one used for all specimens for accurate spatial reconstruction. The data volume was then transferred into a third party software Amira (FEI VSG, Berlin, Germany). In Amira, a volume of interest (VOI) of 100 × 100 × 100 pixels was selected at the center of the lesion in the treatment window from immediate dentin surface into the depth (Figure 1). The VOI was averaged into one plot representing the CT values vs. depth. Lesion parameters LD, ΔZ and surface layer mineral density percentage (SL) were calculated on this plot using a spreadsheet software, considering that sound dentin had a maximum 48% mineral by volume and using standard CT value calibration curve as described in details previously [12,14,17,18] and summarized in Figure 2. The scan filenames were coded so that the image analysis operator was blinded to the treatment group. 

### 2.5. Statistical Analysis

Initially, sample size calculation was done based on one-way ANOVA with four groups with the estimated LD mean and standard deviations of 200 to 400 and 100, respectively (pilot study data) with power (1 − β) of 0.80 at 5% error, which yielded a minimum size of 7 per group. The mean and standard deviation for each group (n = 8) were calculated and data were analyzed using one-way ANOVA with Bonferroni posthoc comparisons (α = 0.05) for LD, ΔZ and SL as variables using in SPSS software version 16 (SPSS Inc., Chicago, IL, USA). 

## 3. Results

After the application of KI to samples containing SDF visual changes were immediately noted. Samples that did not contain KI were colored a dark black, while those samples that received enrichment with KI appeared a more light grey.

After completing data collection a representative mineral density vs. depth profile averaged over all of the specimens, is presented in Figure 2. The control group showed a typical profile of active carious lesion with no surface layer and lowest mineral densities throughout the depth. On the other hand, F-Varnish visually showed higher mineral density values compared to SDF and SDF+KI.

**Figure 3:**
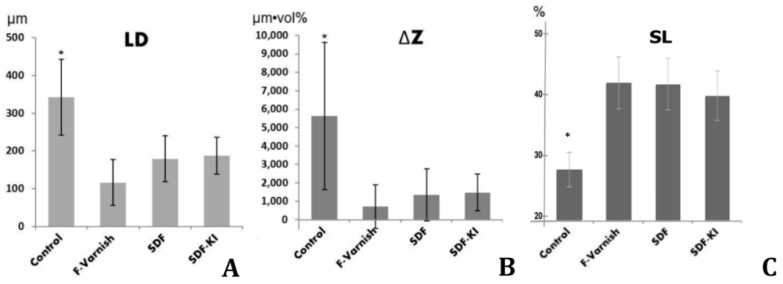
Results of all groups; (**A**) lesion depth (LD); (**B**) mineral loss (ΔZ); (**C**) surface layer mineral density (SL). Asterisk (*) indicates significantly different group in each parameter (P < 0.05).

LD results derived from the mineral density profile of each group are summarized in Figure 3A. The F-Varnish group had the smallest LD and the control had the largest LD. Statistically, there was a significant difference between the control and all other groups (P < 0.001). There was no statistically significant difference among SDF, SDF+KI and F-Varnish (P > 0.05). 

ΔZ results for all groups are summarized in Figure 3B. The ΔZ for each group followed the trend seen in the LD. Although ΔZ values were nominally smaller in F-Varnish than other groups, there was no statistically significant difference among F-Varnish, SDF and SDF+KI; the latter two had similar mineral loss profiles. Again, the control showed the highest ΔZ, which was statistically significantly different from all other groups (P < 0.001).

Results of SL for all groups are summarized in Figure 3C. The surface layer mineral density was similar for all three groups, while the control showed the lowest, significantly different SL (P < 0.001).

## 4. Discussion

KI has been suggested as an additive to alleviate the staining properties of SDF [19,20,21], however, little research has been done to see what effect this additive will have on aspects other than the color. This study showed that in terms of demineralization prevention, there was no significant effect regarding mineral density parameters when compared to SDF by itself. SDF, contains a high amount of fluoride (44,800 ppm), therefore its preventive effects against demineralization could be described by those of F-Varnish (22,600 ppm). In addition, the silver diammine component is reported to inhibit caries formation via inhibition of cysteine cathepsins [22], matrix metalloproteinases [23] and binding to collagen. SDF can increase the microhardness of existing caries lesions [24] and promote remineralization. 

KI solutions have been previously used in dentistry as an antimicrobial agent, and especially used after dental hygiene and scaling [25,26]. These procedures, which often inflame the tissues, can be treated with KI to reduce the damage and the risk of infection. When paired with iodine, KI has been routinely used in endodontics to as an antimicrobial in root canal treated teeth [25], and can also be used as an antifungal agent to treat Candida albicans [26]. KI solution interacts with SDF to form a white-yellow precipitate, which could reduce the amount of free silver ions [27], and form silver iodide. The current findings suggest that SDF+KI showed anti-demineralization properties comparable to those of SDF and F-Varnish. This finding is in line with those of the previous studies [22,28,29], which reported no adverse effects of KI on the anti-caries performance of SDF. On the other hand, a laboratory study [15] that investigated biofilm-induced caries inhibition around glass ionomer restorations reported that SDF+KI was slightly, but statistically significantly, less effective than SDF alone in preventing caries formation.

The null hypothesis in this study was partially rejected as all treatment groups showed different results compared with the control (water). F-Varnish showed good preventive effect against demineralization of the root surface. This effect is thought to be due to the high concentration of fluoride ion in this product, which can protect dentin surface through two chemical mechanisms; neutralizing the local acidity through formation of calcium fluoride in oral plaques and increasing the amount of fluoridated apatite and fluoroapatite in the surface layer, which have higher acid resistance than the non-fluoride variations of naturally occurring dentin apatite. In addition to the chemical effects, sodium fluoride also provides a mechanical one via the base forming a layer on the dentin surface, which may contribute to why it has a strong demineralization resistance. 

Formation of a mineral-rich and acid-resistant surface on tooth surface can improve caries-resistance of the substrate. This study found similar CT values at the surface zone of the dentin treated by SDF and SDF+KI compared to the F-Varnish, leading to the conclusion that SL mineral density was not different among the groups. It could be argued that the presence of metallic silver particles are partly contributing to the radiopacity of the dentin surface in the SDF and SDF+KI groups. This phenomenon was somewhat visible on the internal control windows of the SDF sample presented in Figure 1D(d), suggesting that some particles may have deposited on the adjacent dentin window, even though the SDF solution in these groups were thoroughly rinsed off after application on sound dentin surface, which would remove the majority of free components from the surface. Based on the mineral density profiles, it is reasonable to believe that SDF and SDF+KI worked as well as the F-Varnish in protecting the dentin surface, consistent with previous report on TMR evaluation of SDF-treated dentin [30]. 

On the other hand, some studies [31,32] have emphasized that SDF has advantages over F-Varnish in terms of arresting caries, which have been attributed to its antimicrobial and organic phase properties; however, the current study could not focus on those properties since a bacterial (biofilm-induced) caries model was not employed. In this case, the effect of SDF, and SDF+KI on dentin could be underestimated due to the lack of a natural biofilm. The caries formation with a natural biofilm could yield greater differences between SDF and the varnish, in comparison with the chemical approach of demineralizing dentin as was done in this study. This limitation however, could be alleviated by in vivo studies. It should be noted that pH cycling models such as the one employed in the current study are the most reproducible models for caries simulation laboratory studies [33].

The use of micro CT for this study deserves consideration. TMR has been frequently utilized in past years for analysis of lesions because of its superior resolution using a thin slice of the hard tissue imaged with conventional radiography and microscopy [34]. Standard TMR resolution can range between 5 to 10 µm [35], while micro CT has been shown to be slightly lower in nominal resolution. In the current study, we could consistently image specimens in 3D with voxel resolution of 8.3 µm, which is comparable to the resolution of TMR. It appears that the latest micro CT technology is paving the way for a reliable not destructive tool for mineral content assessment comparable to the gold standard. The non-destructive nature of this method allows for longitudinal studies on the long-term effect of treatments on the mineral content and other attributes of hard tissues.

Clinically, SDF is recommended to be applied at least annually to ensure its effectiveness on root caries [36,37]; the current study demonstrated that a single application of SDF, SDF+KI and F-Varnish could have comparable effects in prevention of root caries.

KI with SDF is already available in some countries as a dental product and its use as a measure to reduce discoloration by SDF is expected to raise [38]. However, clinicians need to be aware of potential drawbacks of adding KI to SDF. For example, microtensile bond strength of dental adhesives decreases considerably, which could affect the ability to place certain resin-based restorations [39]. More studies could be conducted with regard to longevity, technique sensitivity and the reliability of SDF with KI treatment.

## 5. Conclusions

Within the limitations of this laboratory, single application of F-Varnish, SDF and SDF+KI showed comparable preventive effects against root dentin demineralization. Application of KI did not influence anti-demineralization properties of SDF. Micro CT is a quick and effective method for objective and high-resolution characterization of dentin caries lesions.
